# Feasibility and Effectiveness of Recruiting Latinos in *Decídetexto*—A Smoking Cessation Clinical Trial from an Emergency Department Patient Registry

**DOI:** 10.3390/ijerph182010859

**Published:** 2021-10-15

**Authors:** Evelyn Arana-Chicas, Francisco Cartujano-Barrera, Chinwe Ogedegbe, Edward F. Ellerbeck, Lisa Sanderson Cox, Kristi D. Graves, Francisco J. Diaz, Delwyn Catley, Ana Paula Cupertino

**Affiliations:** 1 Department of Surgery and Public Health Sciences, School of Medicine, University of Rochester, Rochester, NY 14642, USA; 2 Department of Public Health Sciences, School of Medicine, University of Rochester, Rochester, NY 14642, USA; Francisco_Cartujano@urmc.rochester.edu (F.C.-B.); Paula_Cupertino@urmc.rochester.edu (A.P.C.); 3 Emergency Trauma Department, Hackensack University Medical Center, Hackensack, NJ 07601, USA; chinwe.ogedegbe@hmhn.org; 4 Department of Medicine and Public Health, University of Kansas Medical Center, Kansas City, KS 66160, USA; EELLERBE@kumc.edu (E.F.E.); lcox@kumc.edu (L.S.C.); 5 Department of Oncology, School of Medicine, Georgetown University, Washington, DC 20007, USA; Kristi.Graves@georgetown.edu; 6 Department of Biostatistics and Data Science, University of Kansas Medical Center, Kansas City, KS 66160, USA; fdiaz@kumc.edu; 7 Department of Pediatrics, Children’s Mercy Hospital, School of Medicine, University of Missouri-Kansas City, Kansas City, MO 64108, USA; dcatley@cmh.edu

**Keywords:** inequality, tobacco use and nicotine dependence, disadvantaged groups and tobacco use

## Abstract

There is an underrepresentation of Latinos in smoking cessation clinical trials. This study describes the feasibility and effectiveness of recruiting Latino smokers in the U.S. from an emergency department (ED) patient registry into a randomized smoking cessation clinical trial. Recruitment occurred from the Hackensack University Medical Center ED. Potential participants were contacted from a patient registry. The primary outcome was whether the participant responded to a call or text. Secondary outcomes included the best day of the week, week of the month, and time of day to obtain a response. Of the 1680 potential participants, 1132 were called (67.5%), while 548 (32.5%) were texted. For calls, response rate was higher compared to text (26.4% vs 6.4%; *p* < 0.001). More participants were interested in the study when contacted by calls compared to text (11.4% vs. 1.8%) and more participants were enrolled in the study when contacted by calls compared to text (1.1% vs. 0.2%). Regression models showed that ethnicity, age, time of day, and week of the month were not significantly associated with response rates. Recruitment of Latinos from an ED patient registry into a smoking cessation clinical trial is feasible using call and text, although enrollment may be low.

## 1. Introduction

Latinos are the largest [1] and second-fastest-growing minority population in the United States [2]. Despite existing disparities in health and healthcare, Latinos are woefully underrepresented in clinical trials [3,4,5,6], comprising only 6% of all oncology clinical trial participants [3]. This limits the generalizability and impact of clinical trial findings to Latinos. Over 6 million U.S. Latino adults (8.8%) are current cigarette smokers [7] and they are more likely to start smoking as they acculturate to the U.S. [8,9]. Latino tobacco use varies based on multiple factors, including country of origin, acculturation to the U.S., and immigration generation [8,9]. For example, while Latinos are less likely to smoke cigarettes and smoke fewer cigarettes per day than non-Latino Whites (NLWs) [8,9], there are differences in smoking prevalence by country of origin, with those of Puerto Rican descent having the highest prevalence (35%), while Dominican individuals have the lowest prevalence (11%) of smoking [10].

There are also notable disparities in tobacco treatment and access [11,12,13,14]. Latino smokers are less likely than NLW smokers to have access to healthcare and resources for smoking cessation, receive advice to quit, and use pharmacotherapy to stop smoking [11,12,13,14]. Only a handful of smoking cessation clinical trials have focused on the cultural and linguistic needs of Latino smokers [15,16,17]. It is important to include more Latinos in smoking cessation clinical trials in order to develop and evaluate effective smoking cessation interventions that take into consideration their smoking patterns and behaviors.

Challenges exist in recruiting Latinos into smoking cessation clinical trials, particularly Latinos who primarily speak Spanish. Some may distrust government-funded research, citing deportation concerns, while others may have never participated in research and distrust the research process [18]. Other barriers to their recruitment may include language, lack of health literacy, and lack of access (e.g., transportation) [4,19,20]. Interestingly, when invited to participate in clinical trials, Latinos enroll at similar rates as NLWs [21,22], indicating they are interested in participating in research when recruitment strategies are culturally and linguistically tailored to them. Although there are studies of various strategies to aid researchers in recruitment for clinical trials (e.g., phone call over mailed letter, tailored messages, and reactive–proactive recruitment phases) [23,24], no study to date has assessed the feasibility and effectiveness of recruiting Latino smokers from an emergency department (ED) patient registry into a smoking cessation clinical trial. Approximately 20% of U.S. Latinos use the ED annually [25] and Latinos are more likely to use the ED for non-urgent or routine care than other racial/ethnic groups [26,27].

The present study describes the feasibility and effectiveness of recruiting Latino smokers from an ED patient registry by call or text message into a randomized clinical trial of *Decídetexto*—a culturally and linguistically tailored mobile smoking cessation intervention designed for Latino smokers [15].

## 2. Materials and Methods

### 2.1. Study Design

This study describes processes and outcomes of ED-based recruitment efforts for a randomized smoking cessation trial, *Decídetexto*. The details of the clinical trial intervention and protocol are described elsewhere [15]. The Institutional Review Board of Hackensack University Medical Center (HUMC) approved this study (#Pro2017-0528).

### 2.2. Setting and Study Population

Recruitment occurred from the HUMC ED in New Jersey, a level II emergency trauma center that sees over 110,000 patients per year in a medically underserved urban community. Over the past 10 years, HUMC’s ED has implemented a program to facilitate enrollment of patients in clinical trials and health services, including smoking cessation [28]. Through this program, HUMC ED patients sign a consent for treatment, which authorizes the healthcare team to give them care as well as to release their medical information to the HUMC care teams to facilitate post-hospital-care treatment and referrals to appropriate health wellness and research programs. Potential participants were identified through the Epic electronic medical record system as Latinos who smoke and were seen at the ED from January 2019 through June 2019 (N = 1680).

### 2.3. Inclusion Criteria

An ED patient registry report was generated by the Business Intelligence (BI) Department at HUMC based on pre-determined eligibility criteria. These criteria included: (1) self-identification as Hispanic or Latino, (2) ≥21 years of age, and (3) self-identification as a current smoker. Exclusion criteria included living outside of Northern NJ counties (e.g., those living outside of Bergen, Essex, Passaic, Paterson, and Hudson counties). Upon receiving the registry, trained research staff members manually excluded patients not residing in Northern NJ counties, who were under age 21, who were duplicated, and who had a clearly incorrect phone number listed (e.g., 999-999-9999).

### 2.4. Recruitment Protocol

Using the registry, research staff contacted potential participants via either calls or texts in June and July 2019. Patients were randomized using the Excel RAND feature to receive either a call or text using a 2:1 allocation ratio, respectively.

Research staff were bilingual (English and Spanish) and trained to use a pre-determined script (in English and Spanish) for calls and texts sent to patients. As previously reported [29], implementing cultural values when recruiting Latinos into research studies is important. The script included Latino values of *personalismo* (warm conversations that convey care and understanding of the patient’s circumstances), *simpatía* (not criticizing the patient), and *confianza* (establishing trust). The phone script was “Good morning, [patient name]. I am calling as part of the medical team at Hackensack Meridian Health (HMH). HMH values the health of our patients. You have been identified as a smoker in your last visit to the emergency room. HMH now offers a smoking cessation service free of charge. We understand that quitting smoking without support is hard and for this reason we would like to offer you help through a free program. Do you have a few minutes to discuss this program?” Due to the fact that the patient registry did not include the preferred language of the patient, research staff adjusted the language during the call. A hospital landline was used to make calls and a study cell phone was used to send text messages.

Texts sent to patients also followed a script and were sent individually using a study cell phone. Our text message scripts were focused on either threat/self-efficacy (e.g., “Smoking is the most important cause of cancer and heart disease. Quitting is possible with help. Enroll today in our free text message program to become an ex-smoker. Reply YES to learn more.”), scarcity (e.g., “Spaces are running out… Enroll today in our free text message program to stop smoking. Reply YES to learn more!”), or social norms (e.g., “Want to stop smoking like thousands of people already did this year? You can do it! Reply YES to learn about our free text message program!”). Patients received one of these three texts. If a participant responded YES or expressed interest, research staff followed up with a phone call. Given that the electronic medical record did not include information on the patients’ language of preference, all text messages were sent in both English and Spanish as one text.

Our bilingual recruitment team underwent multiple mock training sessions using the developed phone call script with other research staff, with one being the recruiter and the other being a patient. One attempt, either call or text, was made at contacting each patient. Research staff tracked the date and time of contacting each patient, and tracked the status of all contacted patients with a code. These codes included: (1) no response, (2) phone disconnected, (3) wrong phone number, (4) left patient voicemail, (5) patient not interested, (6) patient not eligible, (7) research staff spoke with another household member, (8) patient asked to call back later, (9) made patient appointment for enrollment, (10) patient already quit smoking, (11) patient will call us back, (12) patient never smoked, and (13) patient is deceased.

### 2.5. Measures

The primary outcome was whether it was possible to reach this population of Latinos by phone or text (e.g., response vs no response). For the purposes of this study, a “response” was when a participant answered the phone or replied to a text, regardless of interest in participating in the study and/or smoking cessation. Secondary outcomes included the day of the week, week of the month, and time of day to obtain a response. For calls, we divided time of day into four time blocks: 8:30 a.m.–11:29 a.m., 11:30 a.m.–2:29 p.m., 2:30 p.m.–5:29 p.m., and 5:30 p.m.–8:00 p.m. and assessed Monday through Saturday. Per staff availability, calls were not made on Sundays. For text, we divided time of day into two time blocks: 11:30 a.m.–2:29 p.m. and 2:30 p.m.–5:29 p.m. and assessed Monday through Wednesday. These times and days were assessed given the availabilities of research personnel to text participants. Each month of recruitment was divided into four weeks for analysis: Week 1 (days 1–7), Week 2 (days 8–14), Week 3 (days 15–21), and Week 4 (days 22–30/31). Demographic variables included age, gender, and Latino ethnicity.

### 2.6. Analyses

Feasibility of recruitment was defined as the percentage of participants who responded to a call or text. The percentage of responders was calculated separately for call and text and was the number of patient responses out of the total number of patients contacted by either call or text. The effectiveness of recruitment efforts was measured by the percentage of patients who enrolled in our trial. Percent enrolled was calculated separately for call and text and was the number enrolled out of the total number of patients contacted either by call or text.

Data were analyzed using the Statistical Package for Social Sciences (SPSS) version 25 [30]. Overall frequencies and percentages for all categorical variables were calculated, while mean, standard deviation (SD), and range for age were calculated. Bivariate associations between variables were explored using Pearson’s chi-squared test or Fisher’s exact test. The latter was used in instances of one or more of the cells having an expected frequency of five or less [31]. A logistic regression model of the likelihood that participants respond to participation requests through texts vs calls was fitted, adjusting for the variables that were found to be significant in bivariate analyses: age, ethnicity, day of the week, time of day, week of month. Odds ratios (ORs) and 95% confidence intervals (CIs) were calculated. Statistical tests were conducted at a 0.05 significance level and considered to be exploratory. Table 1 and Table 2 show the numbers of subjects included in each analysis. The number of subjects used to calculate a particular statistic may be affected by missing demographics in some subjects and only complete cases were used in the calculation.

## 3. Results

Of 1680 participants from the ED registry who were included in this study, mean age was 40.9 years old (SD:13.8, range: 21–90) (Table 1). Most participants were male (61.3%) and identified as Other Latino (82.7%).

**Figure 1:**
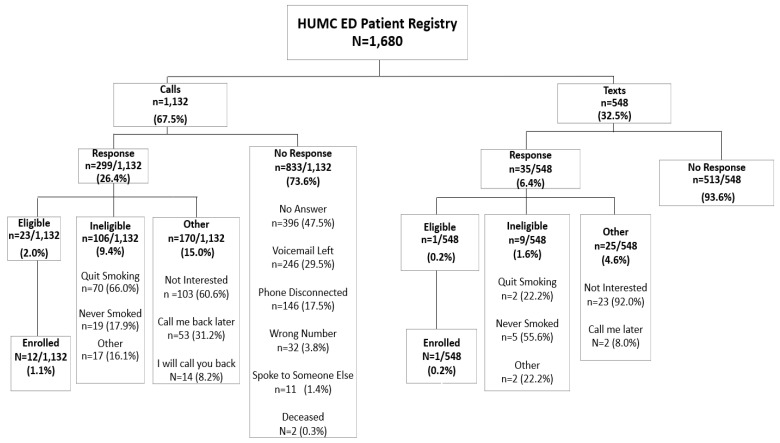
Consort diagram showing the flow of patients that responded to call or text. January 2019–June 2019.

Of the 1680 patients who were identified as Latinos who smoke, 1132 were called (67.5%), while 548 (32.5%) were texted (Figure 1). For calls, response rate was higher compared to text (26.4% vs. 6.4%). A higher rate of participants were interested in the study when contacted by calls compared to text (11.4% vs. 1.8%, as indicated by the number of participants who were eligible or not eligible given that they expressed interest in the study). No participants returned a phone call from a voicemail message left for them. Comparisons of effectiveness showed that a slightly higher rate of participants were enrolled in the study when contacted by calls compared to text (1.1% vs. 0.2%).

Response rate did not differ significantly across gender (*p* = 0.57), even after stratifying for contact method (Table 2). Response rate did not differ by text type either (*p* = 0.90). In the text contact method, response rates differed significantly across age groups in these exploratory analyses (*p* = 0.03). In the call contact method and total sample, response rates differed significantly by day of the week (*p* = 0.01 and *p* < 0.001, respectively). Response rates differed across Latino ethnicities for the text contact method (*p* < 0.001), with Caribbean Latinos having the lowest response rates (2.6%). Overall, calls yielded significantly higher response rates than texts (26.4% vs. 6.4%, *p* < 0.001). In the text group, response rates differed significantly across weeks of the month (*p* = 0.01), with the fourth week yielding the highest response rate.

A logistic regression model ascertained the effect of recruitment contact method on the likelihood that participants respond, adjusting for potential confounding variables identified in Table 2 (Table 3). Participants who were texted had a significantly lower likelihood of responding to participation requests than participants who were called (OR = 0.20; 95% CI, 0.13–0.31). In contrast with bivariate analyses, these adjusted tests showed that ethnicity, age, time of day, and week of the month were not significantly associated with response rates. However, the day of the week was significantly associated with the likelihood of responding to participation requests (*p* = 0.01).

## 4. Discussion

This study contributes to the nascent literature assessing the feasibility and effectiveness of using text messaging and telephone calls to recruit Latino smokers from an emergency department patient registry into a smoking cessation clinical trial, *Decídetexto*. Recruitment by phone call or text message was operationally feasible. Our text response rate was similar to a study by Leavitt et al. that assessed the feasibility of text messages for recruiting pregnant smokers into smoking cessation clinical trials (10.8%) [32]. Our text message enrollment rate was also similar to Leavitt et al.’s study (1.9%). When comparing response rates by call, our study was similar to a study by Hawk et al. that assessed the response rates of phone calls to recruit low-income smokers into a smoking cessation clinical trial (33.0%) [33]. This suggests that it is feasible to reach this population of Latinos who smoke by phone or text through the ED patient registry. While operationally feasible, ED recruitment resulted in low enrollment, despite implementation by a team of diverse and bilingual Latino research staff.

Although approximately a third of patients who responded to a text or call were not interested in our study, Sheppard et al. have shown that Latinos are interested and willing to enroll in clinical trials when invited but the responsibility is on the research team to utilize approaches that facilitate trust and communication (e.g., through partnerships and working with trusted healthcare resources) and incorporating recruitment strategies that are culturally and linguistically tailored to Latinos [23]. Furthermore, studies have shown that the quality of the conversation that occurs to recruit a potential participant is crucial to building participant rapport and their understanding of a clinical trial [34,35]. Despite having a trained and culturally competent recruitment team, our enrollment yield was still low, suggesting this may be necessary but not sufficient for recruitment from an ED patient registry. Research should be done that looks at additional reasons why Latino smokers recruited from an ED patient registry are not enrolling in a clinical trial.

A number of patients on the registry who responded had indicated that they had quit smoking months, sometimes even years, ago, although they were identified in the ED registry as a smoker. There were also patients who indicated never smoking but were in the ED registry as a patient who smokes. It is important that smoking status be assessed at every patient encounter and should consistently be updated in the HMH Epic system. Errors and inaccuracies have been reported to occur in electronic medical record systems [36]. Hospital systems should incorporate methods to more accurately collect patient information.

It is also important to take into consideration the time and resources needed to implement each method. Making phone calls is more time- and resource-consuming than broadcasting a text message. Although in this study we sent text messages individually using a study cell phone, future studies could save time by broadcasting a text to a large group of individuals. Having an identifiable name and phone number appear in the individual’s caller ID could also increase the likelihood of a response. In this study, a phone number appeared in the individual’s caller ID, but no identification of our institution name. Furthermore, future efforts might consider automated telephone calls from an institution to save on resources such as Andrea King’s TelASK Quit Manager, a cloud-based system that interfaces with the electronic health record and manages tobacco cessation programs [37]. Overall, sending text messages may be more cost-effective than making calls and requires less staff to implement. Future studies should consider this cost-effective analysis of texts vs. calls for recruitment.

When looking at the relationships between other covariates and responses to calls and texts, early mornings and evenings appear to be the best time to contact potential participants. Ours is the first study to assess the best time to call for clinical trial recruitment; however, a report assessing the best time of day for job candidate recruitment calls also reported early morning and evening as the best times to receive a response [38]. This may at least partially be due to individuals being less preoccupied during these two times and not being distracted by their current employment responsibilities. Age and gender did not appear to affect the likelihood of a response to call or text. Wednesday and Friday yielded the highest call response rates while Tuesday yielded the highest response rate for text. More research is needed to further determine day(s) of the week that yielded high recruitment rates. Our study found no difference in call response rate by week of the month for calls, yielding different findings from [33] (Hawk et al., 2019), who assessed whether calls by week of the month affects ability to reach low-income smokers and found that the fourth week resulted in the lowest response rate. However, response rates for text messages yielded the highest response rate during the fourth week of the month. Our finding should be interpreted with caution, however, as we were not able to send text messages Thursday through Saturday and the response rate may be significantly different on these dates.

### 4.1. Implications

This study demonstrates that, despite some errors in classification of smoking status, emergency department registries can be used to identify large numbers of smokers in the Latino community. These registries provide a venue for informing these smokers about either clinical or research opportunities to help them quit. While telephone outreach may be more effective than text messaging, response rates to both strategies are modest. This study suggests the need for additional research on ways to improve response rates when communicating with Latino patients in a smoking registry. Such research should consider the complexity of the Latino population (e.g., differences in immigration status, country of origin, language preference, etc.) and incorporate cultural and linguistic methodological approaches.

### 4.2. Limitations and Strengths

The study has several limitations that should be considered when interpreting the findings. Texts were only sent three days per week (Monday–Wednesday) and on two time blocks (11:30 a.m.–2:29 p.m. and 2:30 p.m.–5:29 p.m.). The current study did not complete calls on Sundays; future research should include all days of the week and additional time blocks to assess whether response would differ by call and text. We were unable to collect additional demographic information, including language, health insurance, income, and educational level. The “Other Latino” category as provided by the hospital unfortunately did not include a breakdown of Latino ethnicity, either by country or region. Findings may have been interpreted further with this additional data. It is also possible that the timing and reason for the participant’s ED visit may have impacted their willingness to participate in this study. Lastly, in this study, we only looked at recruitment through the ED. Future studies should assess recruitment through patient registries from other departments (e.g., psychiatry, cardiovascular).

The results of this study are representative of a single contact attempt via call or text. It is likely that the rates of response and enrollment could be increased if multiple attempts were made to contact participants. Future research should assess the effectiveness of recruitment from an ED registry using multiple attempts for both call and text. Moreover, the ED often acts as a safety net for patients with health problems who have no other source of regular care [39,40,41]. It is possible that these individuals are less likely to be able to take advantage of preventive health offerings. Although we did also implement various proactive and reactive recruitment efforts (e.g., health fairs, flyers, radio advertising) for recruitment into *Decídetexto*, those are currently under analysis to be reported in a separate manuscript. This study had representation from different Latino ethnicities, making these results generalizable to other studies aiming to recruit from a diverse group of Latinos from an ED patient registry.

## 5. Conclusions

Recruitment of Latinos from an ED patient registry into a smoking cessation clinical trial is feasible using telephone calls and text messaging, although enrollment may be low. To minimize resource utilization, investigators may focus efforts on enhancing use of text messages via broadcast rather than sending individual text messages. Sending text messages on Tuesdays resulted in the highest response rates and calling or texting in early morning or late evening also resulted in highest response rates. This study found no difference in response rates by week of the month by calls; however, the response rate for texts was highest during the fourth week of the month. Conscious efforts in cultural and linguistic recruitment must be also implemented in order to increase the inclusion of Latinos in clinical trial research.

## Figures and Tables

**Table 1:** Sociodemographic characteristics of the participants by recruitment contact method (N = 1680).

Variable	Call (n = 1132, 67.5%)	Text (n = 548, 32.5%)	Total	*p*-Value
Age (Mean, SD)	41.6 (14.1)	39.4 (13.1)	40.9 (13.8)	0.002
Gender ^a^				0.68
Male	697 (61.6%)	332 (60.6%)	1029 (61.3%)	
Latino ethnicity ^a^				
Central/South American	112 (9.9%)	11 (2.0%)	123 (7.3%)	<0.001
Caribbean ^b^	76 (6.7%)	38 (6.9%)	114 (6.8%)	
Mexican/Chicano	50 (4.4%)	2 (0.4%)	52 (3.1%)	
Other Latino ^c^	893 (78.9%)	497 (90.7%)	1390 (82.7%)	

^a^ The HUMC ED patient registry had some missing demographic data. Thus, the total was not 1680 because of missing data. ^b^ Caribbean included Puerto Ricans and Cubans. ^c^ Hospital records did not collect ethnicity under ‘Other Latino’.

**Table 2:** Bivariate analyses of rates of response to recruitment requests by contact method ^a^.

Variables	Text (n = 548)		Call (n = 1132)		Total (n = 1680)	
	Responded	*p*-Value	Responded	*p*-Value	Responded	*p*-Value
Age		0.03		0.97		0.37
21–44	19/381 (5.0%)		187/704 (26.6%)		206/1085 (19.0%)	
45–69	13/151 (8.6%)		101/388 (26.0%)		114/539 (21.2%)	
70+	3/16 (18.8%)		11/40 (27.5%)		14/56 (4.2%)	
Patient Gender		0.28		0.78		0.57
Male	18/332 (5.4%)		182/697 (26.1%)		200/1029 (19.4%)	
Female	17/216 (7.9%)		117/434 (27.0%)		134/650 (20.6%)	
Latino Ethnicity		<0.001		0.52		0.01
Central/South American	7/11 (63.6%)		30/112 (26.8%)		37/123 (30.1%)	
Caribbean	1/38 (2.6%)		19/76 (25.0%)		20/114 (17.5%)	
Mexican/Chicano	2/2 (100%)		11/50 (22.0%)		13/52 (25.0%)	
Other Latino	25/497 (5.0%)		238/893 (26.7%)		263/1390 (18.9%)	
Day of the Week ^b^		0.18		0.01		<0.001
Monday	7/136 (5.1%)		3/25 (12.0%)		10/161 (6.2%)	
Tuesday	24/296 (8.1%)		78/274 (28.5%)		102/570 (17.9%)	
Wednesday	4/116 (3.4%)		69/211 (32.7%)		73/327 (22.3%)	
Thursday	-		22/84 (26.2%)		22/84 (26.2%)	
Friday	-		58/190 (30.5%)		58/190 (30.5%)	
Saturday	-		69/348 (19.8%)		69/348 (19.8%)	
Time of Day ^c^		0.48		0.63		<0.001
8:30 a.m.–11:29 a.m.	-		54/189 (28.6%)		54/189 (28.6%)	
11:30 a.m.–2:29 p.m.	17/306 (5.6%)		93/376 (24.7%)		110/682 (16.1%)	
2:30 p.m.–5:29 p.m.	17/241 (7.1%)		124/478 (25.9%)		141/719 (19.6%)	
5:30 p.m.–8:00 p.m.	-		28/88 (31.8%)		28/88 (31.8%)	
Week of the Month		0.01		0.72		0.82
Week 1	7/136 (5.1%)		69/269 (25.7%)		76/405 (18.8%)	
Week 2	2/114 (1.8%)		96/349 (27.5%)		98463 (21.2%)	
Week 3	7/147 (4.8%)		66/234 (28.2%)		73/381 (19.2%)	
Week 4	19/151(12.6%)		68/280 (24.3%)		87/431 (20.2%)	
Contact Method						<0.001
Call	-		-		299/1132 (26.4%)	
Text	-		-		35/548 (6.4%)	
Text Type		0.90				
Scarcity	7/180 (3.9%)		-		-	
Social Norms	7/165 (4.2%)		-		-	
Threat/Self- Efficacy	9/186 (4.8%)		-		-	

^a^ Fisher’s exact probability test was used in instances of one or more of the cells having an expected frequency of five or less. ^b^ Thursday, Friday, and Saturday were not included in the analysis given that no texts were sent on these days. ^c^ 8:30 a.m.–11:29 a.m. and 5:30 p.m.–8:00 p.m. were not included in the analysis given that no texts were sent during these times.

**Table 3:** Logistic regression model of responses to recruitment requests.

Variables	Response	
	OR (95% CI)	*p*-Value
Contact Method		<0.001
Call	1.0	
Text	0.20 (0.13–0.31)	
Latino Ethnicity		0.51
Other Latino	1.0	
Central/South American	0.99 (0.49–1.98)	
Caribbean	1.35 (0.64–2.86)	
Mexican/Chicano	0.85 (0.37–1.94)	
Days of the Week		0.01
Monday	1.01 (0.62–1.62)	
Tuesday	1.0	
Wednesday	2.02 (0.91–4.49)	
Thursday	2.13 (0.96–4.72)	
Friday	1.64 (0.62–4.33)	
Saturday	2.11 (0.86–5.19)	
Age		0.52
21–44	1.0	
45–69	0.69 (0.36–1.33)	
70+	0.74 (0.37–1.44)	
Time of Day		0.62
8:30 a.m.–11:29 a.m.	1.24 (0.70–2.17)	
11:30 a.m.–2:29 p.m.	1.28 (0.84–1.97)	
2:30 p.m.–5:29 p.m.	1.0	
5:30 p.m.–8:00 p.m.	1.01 (0.75–1.36)	
Week of Month		0.75
Week 1	0.88 (0.56–1.34)	
Week 2	1.0	
Week 3	0.80 (0.53–1.20)	
Week 4	0.89 (0.55–1.42)	

OR: odds ratio; CI: confidence interval.

## Data Availability

The datasets generated for this study are available on request to the corresponding author.
